# Multi-scale channel attention U-Net: a novel framework for automated gallbladder segmentation in medical imaging

**DOI:** 10.3389/fonc.2025.1528654

**Published:** 2025-01-28

**Authors:** Yiming Zhou, Xiaobo Wen, Kang Fu, Meina Li, Lin Sun, Xiao Hu

**Affiliations:** ^1^ Department of Hepatobiliary Pancreatic Surgery, The Affiliated Hospital of Medical College of Qingdao University, Qingdao, Shandong, China; ^2^ School of Pharmacy, Qingdao University, Qingdao, China; ^3^ Qingdao Cancer Institute, Qingdao University, Qingdao, China; ^4^ Department of Urology, Qingdao Municipal Hospital, Qingdao, Shandong, China; ^5^ Department of ICU, The Affiliated Hospital of Qingdao University, Qingdao, Shandong, China

**Keywords:** deep learning, U-Net, gallbladder, automatically delineated, multi-scale channel attention

## Abstract

**Objectives:**

To develop a novel automatic delineation model, the Multi-Scale Channel Attention U-Net (MCAU-Net) model, for gallbladder segmentation on CT images of patients with liver cancer.

**Methods:**

We retrospectively collected the CT images from 120 patients with liver cancer, based on which ground truth was manually delineated by physicians. The images and ground truth constitute a dataset, which was proportionally divided into a training set (54%), a validation set (6%), and a test set (40%). Data augmentation was performed on the training set. Our proposed MCAU-Net model was employed for gallbladder segmentation and its performance was evaluated using Dice Similarity Coefficient (DSC), Jaccard Similarity Coefficient (JSC), Positive Predictive Value (PPV), Sensitivity (SE), Hausdorff Distance (HD), Relative Volume Difference (RVD), and Volumetric Overlap Error (VOE) metrics.

**Results:**

On the test set, MCAU-Net achieved DSC, JSC, PPV, SE, HD, RVD, and VOE values of 0.85 ± 0.22, 0.79 ± 0.23, 0.92 ± 0.14, 0.84 ± 0.23, 2.75 ± 0.98, 0.18 ± 0.48, and 0.22 ± 0.42, respectively. Compared to the control models, U-Net, SEU-Net and TransUNet, the MCAU-Net improved DSC 0.06, 0.04 and 0.06, JSC by 0.09, 0.06 and 0.09, PPV by 0.08, 0.08 and 0.05, SE by 0.05,0.05 and 0.07, and reduced HD by 0.45, 0.28 and 0.41, RVD by 0.07, 0.03 and 0.07, VOE by 0.04, 0.02 and 0.08 respectively. Qualitative results revealed that MCAU-Net produced smoother and more accurate boundaries, closer to the expert delineation, with less over-segmentation and under-segmentation and improved robustness.

**Conclusions:**

The MCAU-Net model significantly improves gallbladder segmentation on CT images. It satisfies clinical requirements and enhances the efficiency of physicians, particularly in segmenting complex anatomical structures.

## 1 Introduction

According to global cancer statistics in 2022 (1), liver cancer ranks the third leading cause of cancer-related deaths worldwide, with increasingly growing annual incidence and mortality rates in many regions, which necessitates precise treatment for liver cancer to improve patients’ survival rates. Radiotherapy (RT) plays a vital role in the multidisciplinary comprehensive treatment of liver cancer, especially in locally advanced or unresectable cases (2–4). RT uses high-energy rays and precise dose distribution to kill or inhibit tumor growth, thereby extending patients’ survival period and improving their quality of life (5–7).

However, RT effect relies on the precise delineation of the tumor and its surrounding adjacent organs, which ensures the dose concentration on the tumor area, thereby inhibiting tumor growth and reducing radiation exposure to normal tissues and potential side effects (8–10). Presently, the delineation of tumors and their surrounding organs are primarily and manually performed by physicians. This process is time-consuming and susceptible to inter- and intra-observer variability, which poses a tremendous challenge to clinical workflows (11–13). Consequently, automated delineation becomes imperative.

Atlas-based automatic delineation software commonly applied in clinical practice can’t satisfy the need of high-precision segmentation when faced with images with complex structures or diverse variability (14, 15). For instance, the liver and its adjacent organs tend to manifest complex anatomical structures and blurred boundaries on CT images, provoking overwhelming challenges for precise automatic delineation.

In recent years, the continuous development of artificial intelligence facilitated its application in medical image delineation and made significant progress in image segmentation. Many studies have employed deep learning-based automatic segmentation approach to the delineation of organs at risk (OARs) of liver cancer. However, most existing automated delineation methods primarily focus on tumor target volumes and a few key organs, often neglecting the gallbladder (16–18). In the process of RT, the gallbladder, a critical organ adjacent to the liver, is inevitably radiated and excessive radiation doses may cause side effects such as acute cholecystitis and biliary tract injury, seriously affecting the patient’s treatment outcomes and quality of life.

Accurate gallbladder delineation can not only effectively reduce or avoid side effects, but it also facilitates more dose concentration on the tumor target area, thus obtaining superior dose distribution and optimization of the RT plan. Therefore, it becomes extremely essential to develop an efficient and reliable automated method for gallbladder delineation.

Although deep learning has made significant progress in recent years in the domain of medical image segmentation, there still exist some limitations in the existing deep learning models when they cope with complicated CT images of patients with liver cancer. When faced with the complex anatomical relationships between the liver and gallbladder, these models are often limited and susceptible to noise and blurred boundaries, thereby exhibiting unstable segmentation.

To address the above-mentioned issue, this study proposed a novel channel attention module and incorporated it into the U-Net to construct a novel model, Multi-Scale Channel Attention U-Net (MCAU-Net), which was applied to gallbladder segmentation on CT images of the patients with liver cancer. We aimed to satisfy the demand to enhance the quality of RT plans for liver cancer, especially in the aspect of patient safety and treatment efficacy. The implementation of automated gallbladder delineation contributes to reducing physicians’ workload, diminishing manual delineation errors and strengthening delineation consistency and accuracy, thus providing more reliable data for precise RT.

## 2 Materials and methods

### 2.1 Data process

We retrospectively collected CT images of 120 patients with liver cancer and these images were captured from different models of CT scanners (Details were provided in **Supplementary Part 1**). Inclusion criteria are as follows: a). Complete gallbladder was included in the CT images. b). The gallbladder was clearly visible. c). The scans were conducted according to the standard clinical protocol for liver cancer staging. We preprocessed the input images using Hounsfield Unit (HU) value conversion, window width and window level adjustment, and adaptive histogram equalization. Initially, the images were transformed into HU values by the HU value conversion to prepare them for the adjustment of window width and window level. Subsequently, adjustments to the window width and window level were executed specifically for the visibility of the liver and gallbladder regions, enhancing the contrast in these regions and making the key anatomical structures more clearly visible on the images. After that, adaptive histogram equalization was applied to increase the contrast of the region of interest (ROI), further improving the visibility of key anatomical structures and clarifying the boundaries between the gallbladder and liver on the CT image. This allowed the model to more accurately locate and segment the target region. Finally, the images were normalized to a range of 0-1 using a normalization method.

**Figure 1:**
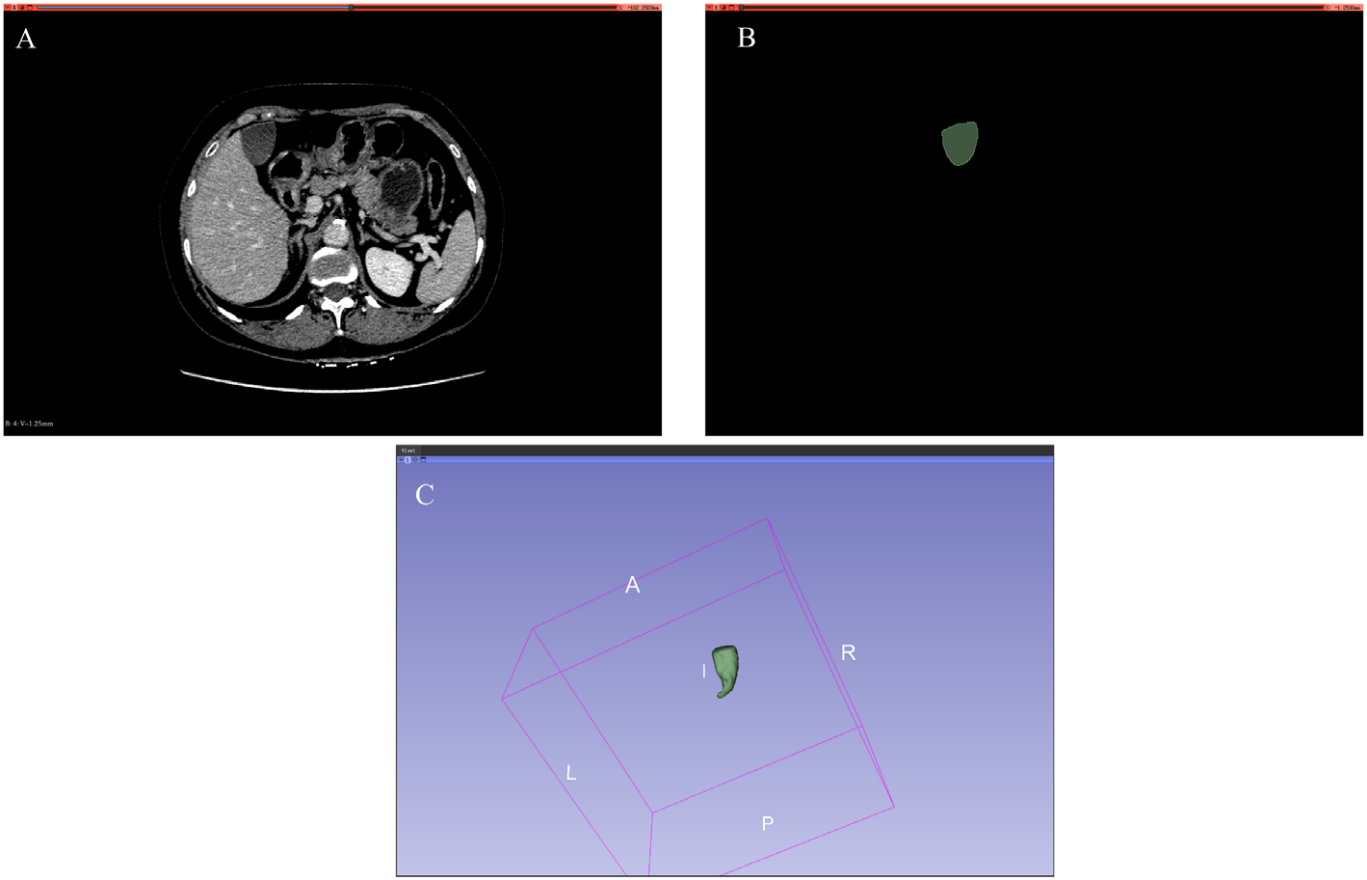
Dataset. **(A)** Input standard image (CT image). **(B)** Corresponding label image (Ground truth). **(C)** Gallbladder drawn in 3D.

We divided the dataset proportionally into a training set (54%), a validation set (6%), and a test set (40%) to effectively evaluate the model’s performance. This kind of division ensures the robustness of the model, avoids overfitting, and provides sufficient data for model evaluation. Furthermore, to address the issue of high costs for data acquisition, data augmentation was performed on the training set, including rotation, shifting, shearing, and zooming, ultimately yielding a total of 7,834 CT images and the dataset is as shown in **Figure 1**.

### 2.2 Module and architecture

Our proposed model evolved from the U-Net model, most commonly used in medical image segmentation, with the features of a symmetrical encoder-decoder architecture, endowing the model with the capability of extracting features layer by layer and efficiently restoring them step by step. The U-Net model also adopts skip connections to compensate lost detailed features in deep networks, thereby effectively improving the segmentation performance.

However, the U-Net model still exists some limitations. First, when handling target regions with complex morphologies and strong background noise, it fails to effectively distinguish the difference between the target region and the background, so when it segments such complex anatomical organs as the liver, the gallbladder region may be segmented mistakenly or overlooked. Additionally, the U-Net model performs unsatisfactorily in capturing multi-scale features. It cannot fully obtain the features of the target region at different scales, which restricts its segmentation of small target regions such as the gallbladder region.

**Figure 2:**
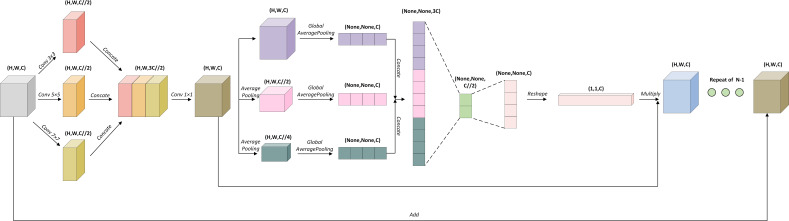
Multi-scale Channel Attention Block (MCA Block).

To address the above-mentioned issues, we have introduced a novel Multi-scale Channel Attention Block (MCA Block) into the U-Net to construct an improved model, MCAU-Net, as shown in **Figure 2**. MCAU-Net is designed to strengthen the U-Net’s ability to capture features at different scales by introducing a channel attention mechanism across different scales to increase the model’s focus on the target area and reduce the interference from background noise. This module falls into two parts: one is a multi-scale feature extraction module used to obtain features of the gallbladder at different scales, and the other is a channel attention module responsible for channel importance re-weight of the input features at different scales.

First of all, the module extracts multi-scale features of the input image through 2D convolutions with kernel sizes of 3, 5, and 7 respectively or 5, 7, and 9 respectively, hence effectively obtaining morphological characteristics of the target region at different scales. Subsequently, these multi-scale features are integrated, and a 1×1 2D convolution is applied to restore the channel dimensions.

Similarly, the channel attention module also employs different scales. It conducts average pooling operations of 2 and 4 on the obtained feature channels to compress the size of the feature maps, thereby extracting more representative global features. After that, global average pooling is performed on both the original input and the feature maps compressed by the average pooling layers, thereby integrating the spatial dimension information into the channel dimension information. Subsequently, the integrated channel information is weighed by cSE, Spatial Squeeze and Channel Excitation attention mechanism, with key features highlighted and irrelevant background information suppressed, whereby the model further improves its segmentation performance in complex backgrounds.

The advantage of the multi-scale channel attention module consists in its ability to fully integrate channel information from different scales, making the model more robust in complex backgrounds. Acquisition and integration of multi-scale features endows the model with stronger efficiency in capturing the gallbladder and its surrounding complex structures. Furthermore, by assigning importance weights to channels at different scales, the multi-scale channel attention mechanism not only concentrates the model’s attention on key features, but also promotes its focus on better correlating feature dependencies between feature maps of different scales.

For the reduction of the model’s parameter count and the costs, the proposed multi-scale channel attention module is only inserted into the downsampling part, that is, replacing the two convolutional operations used for feature extraction with the multi-scale attention module. For the upsampling part, residual connections and Batch Normalization layers are adopted to ensure the stability of model training.

### 2.3 Loss function, evaluation metrics, and baseline models

Class imbalance is a common issue in medical image segmentation, particularly when the target region (such as the gallbladder) occupies a relatively small portion on the entire image.

In the case of class imbalance, the traditional cross-entropy loss function may perform unsatisfactorily because it assigns greater weight to the large number of background pixels (non-target regions), which provokes the model to be more inclined to predict pixels as background. As shown in **Supplementary Part 2**, we calculated the percentage of gallbladder pixels on the total pixels before augmenting the training set and found that the pixels of the gallbladder only accounted for approximately 0.55% of the total, indicating that the gallbladder data can be categorized as extremely imbalanced data.

To address this issue, we adopt Dice Loss as the loss function, which directly measures the overlap proportion between the prediction and the ground truth with more sensitivity to the segmentation of small target regions. The expression for Dice Loss is shown in Equation 1:


(1) $$L_{Dice}=1-\frac{2\sum_{i=1}^{N}p_{i}g_{i}+\overset{`}{\text{o}}}{\sum_{i=1}^{N}p_{i}+\sum_{i=1}^{N}g_{i}+\overset{`}{\text{o}}}$$

where *p_i_* denotes the predicted value of the ith pixel by the model, *g_i_* denotes the true label value of the ith pixel, *N* represents the total number of pixels, and ò is a very small positive number used to avoid a zero denominator.

A series of evaluation metrics, including the Dice Similarity Coefficient (DSC), Jaccard Similarity Coefficient (JSC), Positive Predictive Value (PPV), Sensitivity (SE), Hausdorff Distance (HD), Relative Volume Difference (RVD), and Volumetric Overlap Error (VOE) are used to quantitatively and comprehensively assess the model’s performance. DSC and JSC are overlap-based metrics that measure the similarity between the predicted segmentation and the ground truth, ranging from 0 to 1 with 1 indicating the optimal segmentation. PPV quantifies the proportion of correctly predicted positive pixels, emphasizing the accuracy of the positive predictions, while SE measures the model’s sensitivity to true positive pixels, indicating how effectively the model captures the true segmented regions. HD measures the distance between the farthest points of two irregular shapes and is often utilized to evaluate the boundary segmentation accuracy. RVD evaluates the relative volume difference between the predicted segmentation and the ground truth. The closer the RVD is to 0, the more accurate the segmentation is. Similarly, VOE measures volume overlap error between the predicted segmentation and the ground truth (Formulas are provided in **Supplementary Part 3**).

Besides, we conducted comparative experiments, with three commonly used models in image segmentation, U-Net, SEU-Net, and TransUNet as the control groups, to validate the effectiveness of the proposed MSAU-Net model.

- U-Net (19), the most commonly used model in the field of medical image segmentation, mainly consists of downsampling, upsampling, and skip connections. The downsampling part is responsible for extracting feature information from the target region, while the upsampling part gradually restores the spatial resolution of the image. Through the skip connection mechanism, the features extracted during downsampling are conveyed to the corresponding upsampling layers to compensate for the high-resolution features lost during downsampling, thereby improving segmentation accuracy.
- SEU-Net (20) is an improved model, which is constructed by introducing Squeeze-and-Excitation module into U-Net. The Squeeze-and-Excitation module enhances the model’s focus on important features by recalibrating the relationships between channels. Specifically, the Squeeze-and-Excitation module performs global average pooling on the features of each channel to generate a global receptive field for the channel, and then learns the weight coefficients of each channel through fully connected layers. In this way, the model can automatically learn and emphasize the feature channels that are more contributive to the segmentation, while suppressing irrelevant or interfering information, thereby improving segmentation performance.
- TransUNet (21): TransUNet is a hybrid model that combines the strengths of U-Net and Transformer-based architectures. The model incorporates transformer to improve the model’s ability to capture long-range dependencies and contextual information on medical images. This helps address the limitations of traditional CNN-based architectures, such as U-Net, by enhancing the model’s global receptive field and making it more effective in handling complex structures and diverse medical image modalities.

### 2.4 Model environments and training hyperparameters

The models were constructed using TensorFlow software version 2.4.0 (Google Brain Team,2015; Mountain View, CA, USA) and Keras software version 2.4.3, with Python 3 as the programming language, Windows 10 64-bit as operating system (Microsoft Corp., Redmond, WA, USA), Intel Core i9-10900 KF @ 3.70 GHz (Intel Corp., Santa Clara, CA, USA)as the CPU, NVIDIA GTX3090 24 G (NVIDIA Corp., Santa Clara, CA, USA) as the graphics card, and 128GB memory.

The remaining hyperparameters are listed in **Table 1**. The Batch Size indicates the number of training samples processed before the model’s internal parameters are updated. The Block Number specifies the count of layers or blocks within the network, affecting the model’s depth and complexity in feature extraction. The Epoch count represents how many times the entire dataset is passed through the neural network, crucial for the thorough training of the model. The Learning Rate is vital for determining the step size at each iteration towards minimizing the loss function, while Decay Steps and Decay Rate manage the frequency and scale at which the learning rate is reduced, allowing for finer adjustments and more stable convergence as training progresses.

**Table 1:** Network Training parameters.

Model	Batch Size	Block Number	Epoch	Learning Rate	Decay Steps	Decay Rate
U-Net	4		80	3e-6		
SEU-Net	4		80	6e-5	1200	0.96
TransUNet	4		80	6e-4	1000	0.96
MCAU-Net	4	1	80	4e-4	800	0.96
MCAU-Net	4	2	80	5e-4	800	0.96
MCAU-Net	4	3	80	4e-4	800	0.96

## 3 Results

### 3.1 Module testing

To explore the effect of the diverse number of MCA Blocks on model performance, we performed a series of comparative experiments, where we inserted different numbers of MCA Blocks into the encoder of the U-Net to construct three variant models respectively, named MCAU-Net-1(with 1 MCA block), MCAU-Net-2(with 2 MCA blocks), and MCAU-Net-3(with 3 MCA blocks).

**Figure 3:**
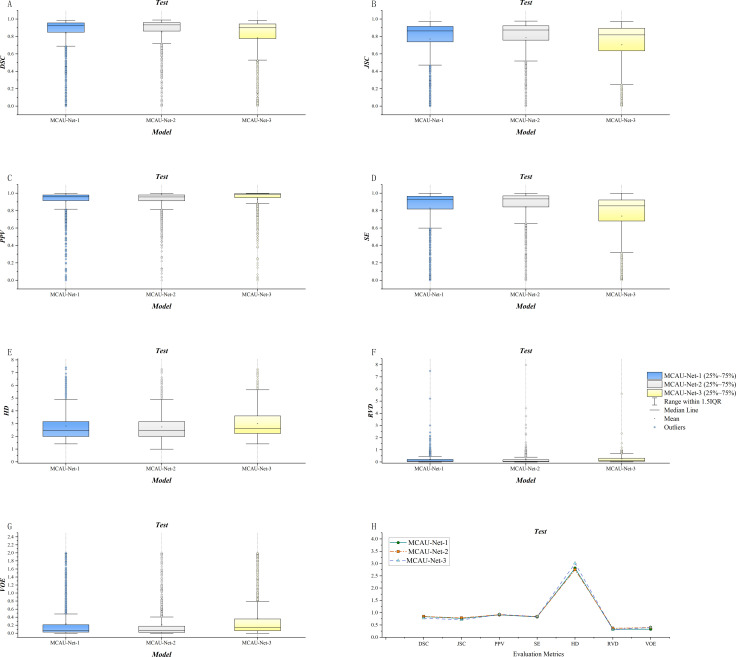
**(A)** Comparison of DSC for the three models. **(B)** Comparison of JSC for the three models. **(C)** Comparison of PPV for the three models. **(D)** Comparison of SE for the three models. **(E)** Comparison of HD for the three models. **(F)** Comparison of RVD for the three models. **(G)** Comparison of VOE for the three models. **(H)** Summary of evaluation metrics (DSC, JSC, PPV, SE, HD, RVD, VOE) for the three models.

The three variants were evaluated respectively using quantitative indicators and the results are presented in **Table 2** and **Figure 3**. The results indicate that MCAU-Net-1 achieved satisfactory DSC and JSC values. Furthermore, MCAU-Net-2 further improved the DSC and JSC, reaching values of 0.85 ± 0.22 (DSC) and 0.79 ± 0.23 (JSC) respectively. This demonstrates the model’s enhanced capability in segmenting the target region.

**Table 2:** Quantitative comparison of different models.

	MCAU-Net-1	MCAU-Net-2	MCAU-Net-3
DSC	0.84 ± 0.23	**0.85 ± 0.22**	0.78 ± 0.27
JSC	0.77 ± 0.24	**0.79 ± 0.23**	0.71 ± 0.27
PPV	0.91 ± 0.16	0.92 ± 0.14	**0.93 ± 0.17**
SE	0.83 ± 0.25	**0.84 ± 0.23**	0.74 ± 0.28
HD	2.81 ± 1.02	**2.75 ± 0.98**	3.01 ± 1.08
RVD	0.20 ± 0.46	**0.18 ± 0.48**	0.25 ± 0.31
VOE	0.23 ± 0.42	**0.22 ± 0.42**	0.36 ± 0.52

The values in bold represent the highest scores achieved.

However, when the number of MCA Blocks increased to 3 (MCAU-Net-3), the model performed less effectively, compared to MCAU-Net-1 and MCAU-Net-2, with only the PPV slightly increasing to 0.93 ± 0.17, but a slight decrease in both the DSC and the JSC and increase in the HD and the VOE,which indicates that the model may experience feature redundancy with too many MCA Blocks, resulting in a slight decrement in segmentation performance.

Overall, MCAU-Net-2 achieved superior performance across multiple indicators, suggesting that moderate increase in the number of MCA Blocks can effectively improve segmentation of the model. However, an excessive number of attention modules and convolutional modules might provoke a slight decline in model performance.

Furthermore, the box plots reveal that MCAU-Net-2 presents smaller interquartile ranges and 1.5IQR and bigger median and mean values across most evaluation metrics. This indicates that MCAU-Net-2 outperforms MCAU-Net-1 and MCAU-Net-3 in terms of model robustness and generalization, and provides relatively ideal segmentation performance while maintaining low computational complexity.

### 3.2 Ablation study of multi-scale feature extraction block

To identify the optimal multi-scale levels for the multi-scale feature extraction block, we conducted further ablation experiments. To explore the impact of different convolution kernel sizes on model performance and memory consumption, we tested two combinations of different convolution kernel sizes: 3, 5, 7 and 5, 7, 9.

As illustrated in **Table 3**, the combination of kernel sizes 3, 5, 7 demonstrated superior performance compared to that of kernel sizes of 5, 7, 9 across all evaluated metrics, among which the DSC for the combination of 3, 5, 7 was 0.85 ± 0.22, better than a DSC of 0.83 ± 0.24 for the combination of 5, 7, 9. Additionally, the JSC (0.79 ± 0.23) for the combination of 3, 5, 7 was also higher than 0.76 ± 0.25, the JSC for the combination of 5,7,9, suggesting a higher accuracy in gallbladder segmentation. The PPV for the combination of 3, 5, 7 was 0.92 ± 0.14, which was higher than 0.90 ± 0.17, the PPV for the combination of 5,7,9. This also indicates that the combination of 3, 5, 7 is more effective in reducing false positives in non-gallbladder regions.

**Table 3:** Ablation study of different kernel size.

	Kernel size (3,5,7)	Kernel size (5,7,9)
DSC	**0.85 ± 0.22**	0.83 ± 0.24
JSC	**0.79 ± 0.23**	0.76 ± 0.25
PPV	**0.92 ± 0.14**	0.90 ± 0.17
SE	**0.84 ± 0.23**	0.82 ± 0.25
HD	**2.75 ± 0.98**	2.90 ± 1.03
RVD	**0.18 ± 0.48**	0.20 ± 0.47
VOE	**0.22 ± 0.42**	0.25 ± 0.45

The values in bold represent the optimal values achieved.

As for boundary fitting capability, HD for the combination of 3, 5, 7 was 2.75 ± 0.98, which was lower than 2.90 ± 1.03, HD for the combination of 5, 7, 9, reflecting more precise fitting to the gallbladder boundary. Additionally, SE also showed a slight improvement, with a value of 0.84 ± 0.23 for the combination of 3, 5, 7, a bit higher than 0.82 ± 0.25 for the combination of 5, 7, 9. This indicates that the combination of 3, 5, 7 has an enhanced coverage rate and reduced detection omission in capturing gallbladder region features. Similarly, RVD and VOE presented improvements with values of 0.18 ± 0.48 vs. 0.20 ± 0.47 and 0.22 ± 0.42 vs. 0.25 ± 0.45, respectively, reflecting a more accurate volume measurement with less discrepancy and fewer overlap errors.

Overall, the experimental results demonstrate that the smaller kernel size combination of 3, 5, 7 exhibits higher segmentation accuracy, lower false positive rates, and better boundary fitting capability in gallbladder segmentation tasks. Additionally, smaller kernel sizes means fewer parameters, decreasing the computational burden.

### 3.3 Ablation study of different modules

To further evaluate the individual and synergistic contributions of the multi-scale feature extraction and channel attention mechanisms in the MCAU-Net model, we conducted a series of ablation studies. The ablation experiments were designed to quantitatively assess the impact of each module on segmentation performance, thereby validating the necessity and effectiveness of integrating these components. In the ablation study, we selected the number of each module to be 2. As shown in **Table 4**, embedding either multi-scale feature extraction or the channel attention mechanism alone can improve model performance, but the best results are achieved when both modules are incorporated. Specifically, the MCA Block achieves optimal values across all metrics. These results demonstrate that the combination of multi-scale feature extraction and multi-scale channel attention can significantly enhance the overall segmentation performance and play a crucial role in gallbladder segmentation. Therefore, integrating these two mechanisms is essential for improving the model’s generalization ability and segmentation effectiveness.

**Table 4:** Ablation study of different modules.

	Baseline	Multi-scale Feature Extraction Block	Multi-scale cSE Block	MCA Block
DSC	0.79 ± 0.23	0.83 ± 0.24	0.84 ± 0.23	**0.85 ± 0.22**
JSC	0.70 ± 0.24	0.75 ± 0.25	0.77 ± 0.24	**0.79 ± 0.23**
PPV	0.84 ± 0.20	0.90 ± 0.18	0.92 ± 0.15	**0.92 ± 0.14**
SE	0.79 ± 0.24	0.80 ± 0.26	0.82 ± 0.24	**0.84 ± 0.23**
HD	3.20 ± 1.02	2.87 ± 1.05	2.84 ± 1.03	**2.75 ± 0.98**
RVD	0.25 ± 0.53	0.21 ± 0.57	0.19 ± 0.45	**0.18 ± 0.48**
VOE	0.26 ± 0.38	0.26 ± 0.46	0.24 ± 0.43	**0.22 ± 0.42**

The values in bold represent the optimal values achieve. (The models in the ablation study were U-Net).

### 3.4 Comparison of binary cross entropy and dice loss

**Figure 4:**
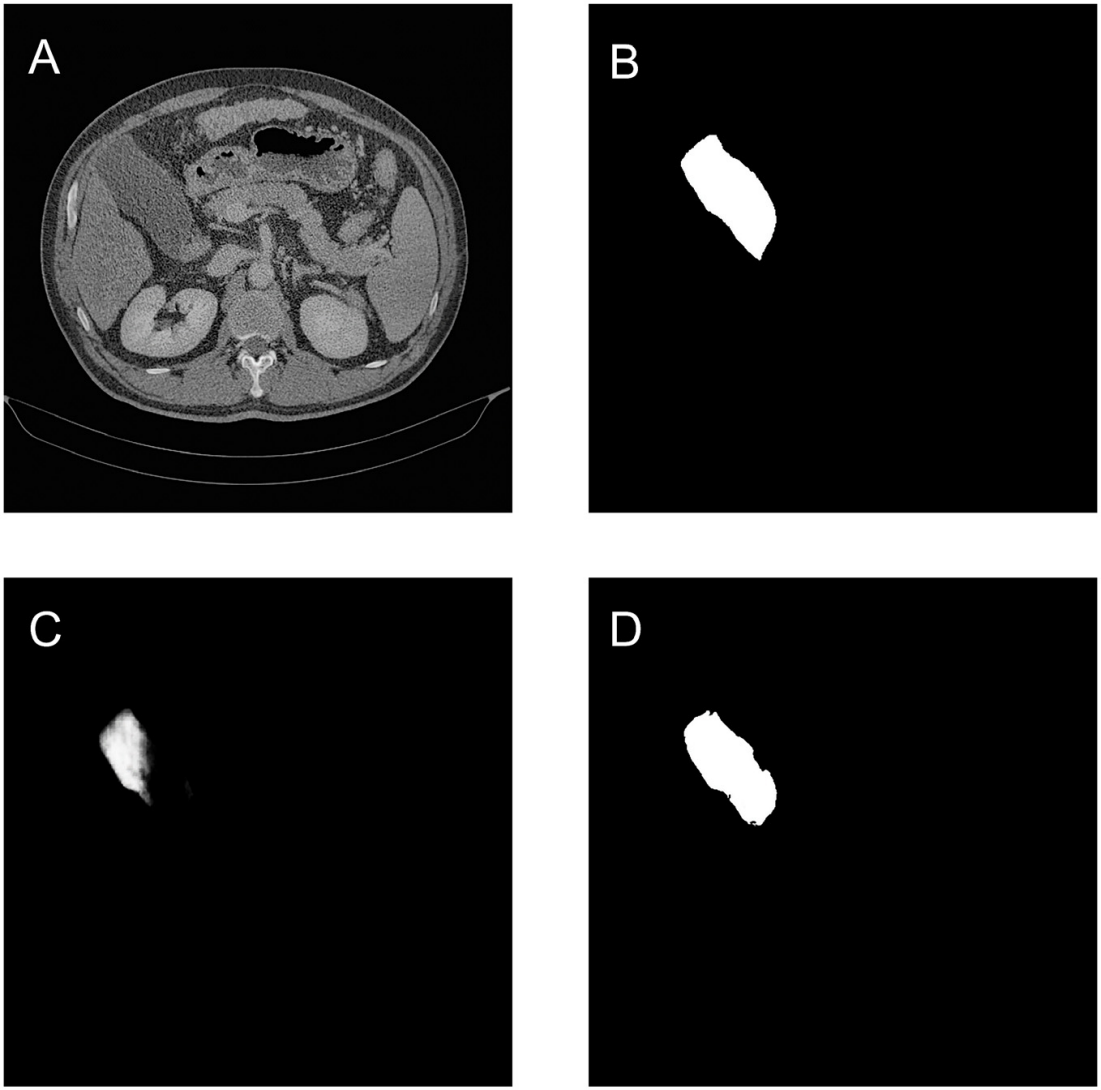
Gallbladder segmentation by MCAU-Net based on BCEL and on DL. **(A)** CT image. **(B)** Ground truth **(C)** Segmentation by MCAU-Net based on BCEL **(D)** Segmentation by MCAU-Net based on DL.

As shown in **Table 5**, the quantitative results demonstrate that the MCAU-Net model performs better based on Dice loss (DL) than on the Binary Cross Entropy Loss (BCEL) across all metrics, with significant improvements. Specifically, DSC improved from 0.80 ± 0.20 (BCEL) to 0.85 ± 0.22 (DL), JSC from 0.71 ± 0.22 (BCEL) to 0.79 ± 0.23(DL), PPV from 0.83 ± 0.21(BCEL) to 0.92 ± 0.14 (DL), SE from 0.81 ± 0.22(BCEL) to 0.84 ± 0.23(DL); HD lowered from 3.22 ± 1.02(BCEL) to 2.75 ± 0.98 (DL), RVD from 0.29 ± 0.67 (BCEL) to 0.18 ± 0.48 (DL) and VOE from 0.25 ± 0.31(BCEL) to 0.22 ± 0.42(DL). Qualitative results (as illustrated in **Figure 4**) reveal that the MCAU-Net model based on BCEL exhibits blurring in the segmented image possibly because of BCEL’s limitations when dealing with class imbalance. Since the background typically occupies a larger proportion, the BCEL tends to prioritize the classification of background pixels during training, posing less accurate discrimination of the target regions and manifesting as blurriness.

**Table 5:** Comparison of binary cross entropy loss and dice loss function.

	BCEL	DL
DSC	0.80 ± 0.20	**0.85 ± 0.22**
JSC	0.71 ± 0.22	**0.79 ± 0.23**
PPV	0.83 ± 0.21	**0.92 ± 0.14**
SE	0.81 ± 0.22	**0.84 ± 0.23**
HD	3.22 ± 1.02	**2.75 ± 0.98**
RVD	0.29 ± 0.67	**0.18 ± 0.48**
VOE	0.25 ± 0.31	**0.22 ± 0.42**

The values in bold represent the optimal values achieve.

### 3.5 Quantitative results

We chose the MCAU-Net 2(Here after MCAU-Net), the best among the three variants, to be compared with U-Net, SEU-Net and TransUNet. **Table 6** presents the quantitative results of U-Net, SEU-Net, TransUNet, and MCAU-Net on multiple segmentation evaluation metrics. The MCAU-Net outperforms U-Net, SEU-Net and TransUNet on most evaluation metrics. The MCAU-Net increased the DSC by 0.06, 0.04 and 0.06 respectively, and the JSC by 0.09, 0.06 and 0.09 respectively compared to U-Net, SEU-Net and TransUNet, indicating that MCA block greatly enhances the model’s ability to accurately capture the target region.

**Table 6:** Quantitative comparison of different models.

	U-Net	SEU-Net	TransUNet	MCAU-Net
DSC	0.79 ± 0.23	0.81 ± 0.21	0.79 ± 0.25	**0.85 ± 0.22**
JSC	0.70 ± 0.24	0.73 ± 0.23	0.70 ± 0.25	**0.79 ± 0.23**
PPV	0.84 ± 0.20	0.84 ± 0.21	0.87 ± 0.20	**0.92 ± 0.14**
SE	0.79 ± 0.24	0.79 ± 0.23	0.77 ± 0.27	**0.84 ± 0.23**
HD	3.20 ± 1.02	3.03 ± 1.03	3.16 ± 1.07	**2.75 ± 0.98**
RVD	0.25 ± 0.53	0.21 ± 0.44	0.25 ± 0.55	**0.18 ± 0.48**
VOE	0.26 ± 0.38	0.24 ± 0.38	0.30 ± 0.46	**0.22 ± 0.42**

The values in bold represent the optimal values achieve.

Additionally, MCAU-Net significantly improved PPV and SE, achieving values of 0.92 ± 0.14 and 0.84 ± 0.23, respectively. These enhancements indicate the model’s accurate prediction and comprehensive detection capabilities for positive sample regions. Meanwhile, the reduction in HD by MCAU-Net suggests its better performance than the control group and better spatial consistency in reproducing the boundaries of the target region, with segmentation closer to the true boundaries.

### 3.6 Qualitative results

**Figure 5:**
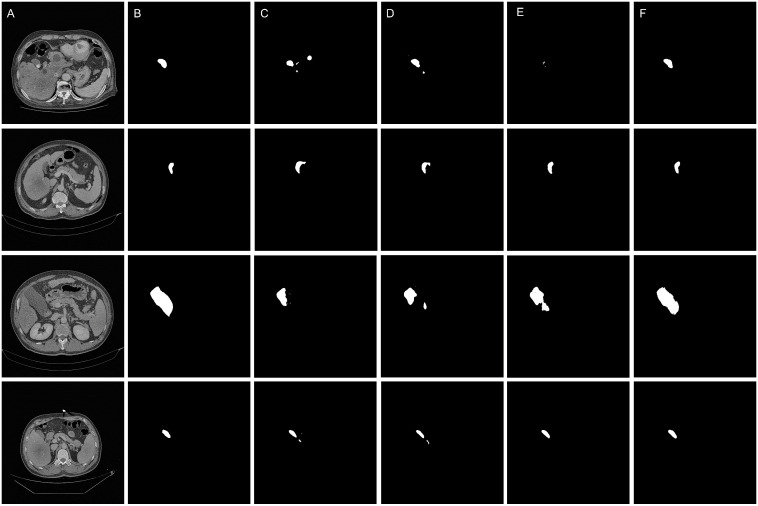
Qualitative results of the segmentation models. **(A)** Original images. **(B)** Ground truth **(C)** Segmentation by U-Net model **(D)** Segmentation by SEU-Net model **(E)** Segmentation by TransUNet model **(F)** Segmentation by MCAU-Net model.

**Figure 6:**
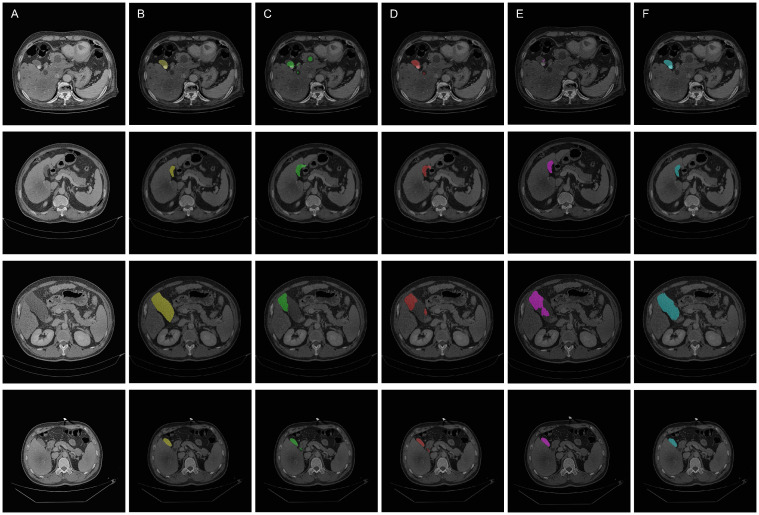
Qualitative overlay results of the segmentation models. **(A)** Original images. **(B)** Ground truth **(C)** Segmentation by U-Net model **(D)** Segmentation by SEU-Net model **(E)** Segmentation by TransUNet model **(F)** Segmenation by MCAU-Net model.

The qualitative results are shown in the **Figures 5** and **6**, indicating that the U-Net model (Column C) can roughly identify the target area, but it exhibits deficiencies in capturing edge details and recognizing the scope of the target area, making it prone to over-segmentation, under-segmentation, and scattered points.

Comparatively, the SEU-Net, as shown in **Figures 5** and **6** (Column D), has presented improved focus on the target area, strengthened suppression of background noise, and relatively reduced scattered points and over-segmentation/under-segmentation. However, the SEU-Net still exhibits some scattered points where there are complex structures around the gallbladder. Additionally, when dealing with larger areas (the third row of figures), the model struggles to outline complete contours, leading to less accurate segmentation results.

TransUNet exhibits certain improvement in the detailed edge segmentation and large region procession, as shown in **Figures 5** and **6** (Column E). This may be attributable to the introduction of the Transformer mechanism, which can better capture global information and features of edge details. However, TransUNet has provoked no significant segmentation improvements and still presents obvious under-segmentation and notable deficiencies when processing large regions.

By contrast, the MCAU-Net, as depicted in **Figures 5** and **6** (Column F), delineates the boundary of the target area more accurately, decreases the scattered points, and fully identifies the structural contours of larger areas with more similarity to the ground truth. Overall, the MCAU-Net significantly outperforms U-Net, SEU-Net and TransUNet in terms of segmentation accuracy and robustness, and demonstrates higher accuracy and completeness particularly when dealing with complex structures and larger target areas.

### 3.7 Box plots

**Figure 7:**
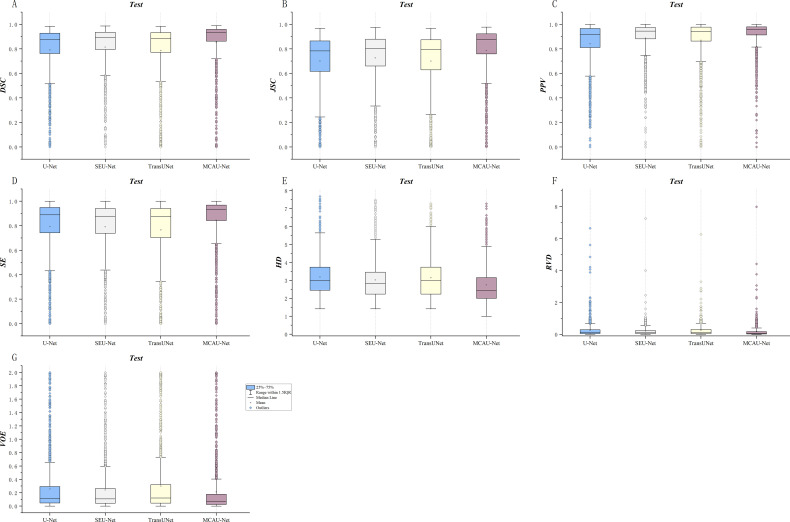
Quantitative comparison of segmentation by the four models across various metrics. **(A)** DSC comparison **(B)** JSC comparison **(C)** PPV comparison **(D)** SE comparison. **(E)** HD comparison **(F)** RVD comparison. **(G)** VOE comparison.

The boxplots in the **Figure 7** show the segmentation performances of different models. As illustrated in **Figure 7**, the MCAU-Net obtained a significantly higher median and mean in most evaluation metrics compared to U-Net, SEU-Net and TransUNet, indicating its significant advantage in segmentation accuracy. This reveals that MCAU-Net effectively enhances the recognition ability for target areas, especially performing excellently in complex backgrounds and boundary detail segmentation. Moreover, the smaller interquartile range of MCAU-Net implies that its segmentation performance fluctuates less across different image samples, demonstrating higher stability.

In summary, the box plots demonstrate that MCAU-Net significantly outperforms U-Net, SEU-Net and TransUNet in terms of segmentation accuracy, performance stability, and consistency. These advantages ensure that MCAU-Net delivers higher segmentation quality and reliability in complex medical image segmentation, fully validating the effectiveness and practicality of the proposed MCA block in enhancing model performance.

### 3.8 Parameter comparison and inference time

The comparison of parameters number, FLOPs (Floating Point Operations Per Second) and inference time among U-Net, SEU-Net, TransUNet, and MCAU-Net (Shown in **Table 7**) reflects the computational complexity of each model.

**Table 7:** Comparison of parameter counts and FLOPS(M).

	U-Net	SEU-Net	TransUNet	MCAU-Net
Total params	1,940,817	1,986,505	103,239,195	3,507,705
Trainable params	1,940,817	1,985,481	95,786,331	3,504,281
FLOPS(M)	9.66e+04	1.12e+05	2.77e+05	2.01e+05
Inference Time(s)	77.92	79.98	168.88	100.47

In terms of FLOPs, MCAU-Net requires 2.01e+05 M computations, higher than that of U-Net (9.66e+04 M) and SEU-Net (1.12e+05 M), indicating that MCAU-Net demands increased computation. However, these additional parameters and computational costs contribute to better model performance, as illustrated by the quantitative results in the previous chapters. In contrast, TransUNet requests the highest FLOPs value: 2.77e+05 M, but its segmentation performance shows either limited improvement or, in some cases, a slight decline compared to MCAU-Net. This observation suggests that the increased computational demand of TransUNet does not proportionally result in improved segmentation performance. MCAU-Net, on the other hand, demonstrates a more efficient utilization of computational resources, yielding superior performance with a lower computational burden compared to transformer-based architectures.

Regarding inference time, MCAU-Net’s inference time is 100.47 seconds, slightly longer than 77.92 (U-Net’s) and 79.98 seconds (SEU-Net’s), but still significantly lower than 168.88 seconds (TransUNet’s). This indicates that MCAU-Net not only outperforms Transformer-based architectures in terms of computational complexity, but also achieves better segmentation results with relatively lower inference time. For real-time or near-real-time clinical needs, MCAU-Net’s inference efficiency holds high practical value.

Although its inference time is a little longer than that of U-Net and SEU-Net, MCAU-Net exhibits better segmenation. It’s suggested that MCAU-Net may be more suitable for scenarios where quick and precise decision-making is crucial, such as clinical settings where rapid analysis of medical images is required. The ability to maintain a balance between segmentation accuracy and inference speed makes MCAU-Net a promising candidate for various medical image analysis tasks.

MCAU-Net demonstrates a good balance between model complexity and segmentation effectiveness, providing superior accuracy and robustness in the segmentation of complex anatomical structures such as the gallbladder. This comparison highlights the trade-off: although MCAU-Net involves a higher computational load, its enhanced architecture brings significant improvements in segmentation quality, making it particularly suitable for clinical scenarios where high precision is required.

### 3.9 Analysis of failure cases

**Figure 8:**
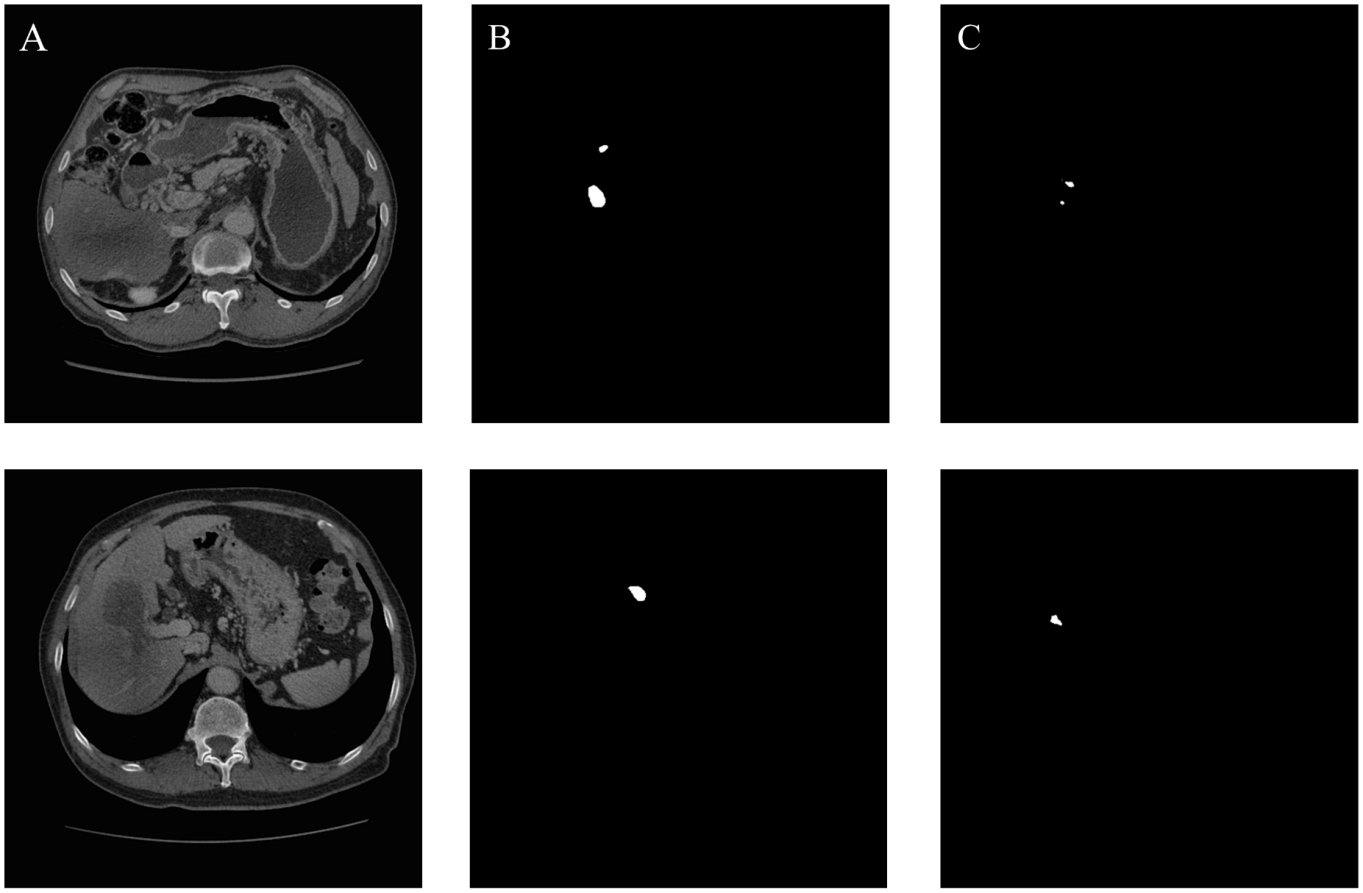
Failure cases analysis **(A)**. CT image **(B)**. Ground Truth **(C)**. Segmentation by MCAU-Net.

Despite the overall excellent performance, our proposed models still presents significant prediction biases in certain specific scenarios. To explore the potential factors accounting for the failed cases, we analyzed gallbladder segmentation images from the test set that exhibited poor qualitative results. As shown in **Figure 8**, the model is more prone to missing or misclassifying segmentation when the gallbladder volume is extremely small with surrounding tissues that have extremely similar gray level or morphology to the gallbladder, or whose gray level distribution is complex. This might be attributed to the limitations of the attention mechanism in feature extraction of extremely small targets and scenarios with complex surrounding structures. Additionally, such samples account for a relatively low proportion in the training data, challenging the model’s robustness and generalization. Future research may further promote the robustness of the model in various complex scenarios by including more diverse samples and adopting such strategies as Adaptive Saliency Detection.

## 4 Discussion

Deep learning-based automatic segmentation is rapidly prevailing in medical image segmentation and becoming a major research focus, and it has exhibited significant potential especially in complex tasks such as tumor and organ segmentation.

Compared to traditional manual segmentation, deep learning models, with the support of large-scale datasets, can automatically extract image features, significantly improve segmentation efficiency and ensure a certain degree of consistency and objectivity, thereby reducing subjective and inter-and- intra -observer variability. Despite the outstanding performance of deep learning technology in the field of automatic segmentation, there still exist numerous challenges, especially when dealing with multi-scale, fine structures, and complex background noise, where the accuracy and robustness of the model may be limited.

Through introducing a plug-and-play MCA Block, our proposed model, MCAU-Net, significantly enhances segmentation performance while maintaining the simplicity of the U-Net structure.

MCA Block integrates multi-scale feature extraction and multi-scale channel attention mechanisms, which promotes the model’s ability to capture feature information at different scales. The results of multi-scale ablation experiments revealed that the proposed multi-scale network demonstrated consistent convergence and generalizability, effectively segmenting various gallbladder structures on the images, including challenging scenarios with complex anatomical boundaries. Furthermore, by effectively weighting the feature channel information of different sizes and highlighting key channel features, it better correlates the dependencies between feature maps of different scales, which produces higher accuracy and robustness in segmentation, especially in small target areas and complex backgrounds. The experimental results indicated that the MCAU-Net not only maintained high segmentation accuracy, but it also effectively alleviated the issues such as scattered points and failure to segment large areas in the control group models. This improvement resulted from the full capture of multi-scale information as well as benefited from the effective weighting of the multi-scale channel attention mechanism and the correlation between feature maps of different scales. It enhanced long-distance dependency relationships, thereby solving the problem of under-segmentation in large areas.

When DSC is greater than 0.7, the results are regarded acceptable (22–25). Our proposed MCAU-Net model achieved a DSC of 0.85 in experiments, significantly surpassing the benchmarks in this field. The significant improvement validated our model’s superiority in gallbladder segmentation and its reliability and applicability in clinical settings. We further compared our model with some currently proposed models in gallbladder segmentation. Salimi et al. (26) developed a residual network, HighRes3DNet, for organ segmentation in CT images. Their results revealed that the average DSC and JSC values for the gallbladder were 0.79 ± 0.2 and 0.69 ± 0.21, respectively. In comparison, our proposed MCAU-Net achieved a higher DSC value of 0.85, indicating a significant improvement in segmentation accuracy and demonstrating its superior segmentation performance.

Likewise, Shen et al. (27) proposed a deep learning model based on spatial attention and deformable convolution for multi-organ segmentation in abdominal CT images, their proposed model achieved certain improvements in the gallbladder segmentation, with DSC, JSC, and HD values of 0.8046, 0.7036, and 22.97, respectively. In comparison, our proposed MCAU-Net has achieved superior performance on these metrics, with further improvements in DSC and JSC, and a further decrease in HD value. These improvements not only demonstrated MCAU-Net’s higher accuracy and robustness in segmentation precision and boundary description, but also validated its reliability and effectiveness in dealing with complex anatomical structures and suggest its enhanced potential for clinical applications.

Lin et al. (28) proposed a V-Attention U-Net model for the segmentation of OARs in abdominal images (such as the gallbladder). Their results revealed that the V-Attention U-Net achieved DSC of 0.7595 ± 0.1925 and HD of 7.21 ± 13.03 in gallbladder segmentation. Compared to that, the MCAU-Net model proposed in this study exhibited superior performance in segmentation accuracy and boundary description, achieving a higher DSC and a lower HD value. This fully demonstrated the powerful performance of MCAU-Net in gallbladder segmentation. These comparisons further validated MCAU-Net’s advantages in dealing with complex anatomical structures and improving segmentation consistency, making it more potential for clinical applications.

However, this study also has certain limitations. First, the introduction of multi-scale feature extraction has increased the computational requirements and memory consumption of the model to some extent despite the significant progress made by MCAU-Net in multiple aspects. Therefore, future research will focus on exploring more lightweight model designs to reduce computational complexity and enhance the operability and practicality of the model in clinical applications. Second, the model in this study adopts a single-modal design (based on CT images), which may limit its generalization in multi-modal medical images (such as MRI, ultrasound, etc.). As such, future work will consider extending the model to multi-modal data to fully utilize the complementary information provided by different imaging techniques, further improving segmentation performance and model applicability. Third, despite the overall satisfactory performance of our model in gallbladder segmentation, there are still some samples with poor segmentation results, which might be attributed to the limitations of the attention mechanism in feature extraction for extremely small samples, especially when there are numerous complex and similar structures surrounding the target. Furthermore, the insufficient number of extremely small samples in the training set provokes the model’s difficulty in ensuring perfect robustness and generalization in such scenarios. In the future study, we will focus on addressing these limitations, which will help promote the dissemination and application of MCAU-Net in a wider range of clinical scenarios.

In summary, MCAU-Net provides an efficient, robust, and practical solution for automatic gallbladder segmentation. Its advantages in segmentation accuracy, boundary description, and handling of complex structures have the potential to be extended to other medical image segmentation tasks, thereby further advancing the intelligentization of medical image segmentation and providing reliable technical support for clinical diagnosis and treatment. In the future, we will continue to optimize model performance, reduce computational complexity, and expand its scope of application to achieve broader clinical use.

## Data Availability

The datasets generated and/or analysed during the current study are not publicly available due to protection of patient privacy but are available from the corresponding author on reasonable request.
