# Supplementary material for: Multi-scale channel attention U-Net: a novel framework for automated gallbladder segmentation in medical imaging
Source: Front Oncol. 2025 Jan 28;15:1528654. doi: 10.3389/fonc.2025.1528654 (PMC11810919; doi:10.3389/fonc.2025.1528654)
Supplement: Supplementary file 1 [file DataSheet1.docx]

**Multi-Scale Channel Attention U-Net: A Novel Framework for Automated Gallbladder Segmentation in Medical Imaging**

*Yiming Zhou^1^, Xiaobo Wen^2,3^, Kang Fu^1^, Meina Li^4^, Lin Sun^5^, Xiao Hu^1^**

1. *Department of Hepatobiliary Pancreatic Surgery, The Affiliated Hospital of Medical College of Qingdao University, Qingdao, Shandong, China*
2. *School of Pharmacy, Qingdao University, Qingdao, China*
3. *Qingdao Cancer Institute, Qingdao University, Qingdao, China.*
4. *Department of Urology, Qingdao Municipal Hospital, Qingdao, Shandong, China*
5. *Department of ICU,The Affiliated Hospital of Qingdao University, Qingdao, Shandong, China*

*Correspondence to: Dr. Xiao Hu, Email:* ***8371270@qq.com***

**Supplementary Materials**

1. **CT scanners**

In this study, we utilized CT images from various models of CT scanners, including Siemens SOMATOM DEFINITION FLASH, GE Revolution CT (256-slice), GE Optima CT660, and GE Optima CT620. By forming our dataset based on the data from different models of CT scanners, we introduced variability in imaging conditions, which contributes to enhancing the model's generalizability and robustness.

1. **Gallbladder proportion**

As illustrated in Figure 1, the proportion of the gallbladder on the images is mostly close to 0, significantly lower than the threshold, which indicates that the gallbladder occupies a very small portion of the images. Such a significant class imbalance, where the model tends to predict the target (gallbladder) as the background, may pose challenges to the training of the semantic segmentation model.


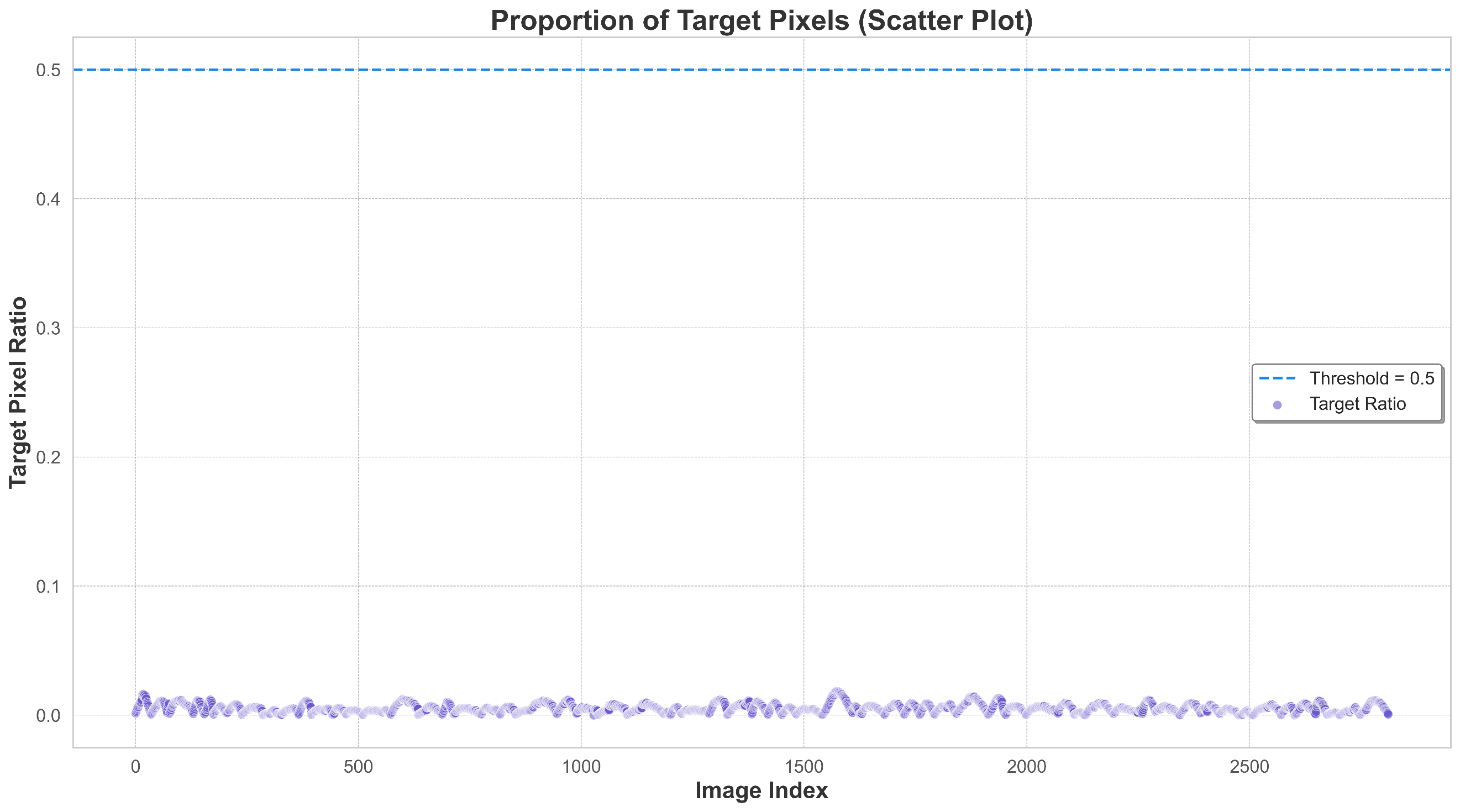


**Figure 1. Gallbladder proportion (The dashed blue line represents the threshold.)**

**3. Evaluation Metrics for Segmentation Accuracy and Generalization**

A series of evaluation metrics, including the Dice Similarity Coefficient (DSC), Jaccard Similarity Coefficient (JSC), Positive Predictive Value (PPV), Sensitivity (SE), Hausdorff Distance (HD), Relative Volume Difference (RVD), and Volumetric Overlap Error (VOE) are used to quantitatively and comprehensively assess the model's performance. The formulas for these metrics are shown in Equation 1-7.

 (1),

 (2),

 (3),

 (4),

 (5),

 (6),

 (7),

where$p_{i}$ denotes the predicted value of the ith pixel, $g_{i}$ denotes the true label value of the ith pixel, N is the total number of pixels and *A* represents the predicted segmentation boundary while *B* denotes the true annotated segmentation boundary.

The value of DSC ranges from 0 to 1, and the closer the value is to 1, the better the model prediction is and vice versa. It's the same with the value of JSC. The smaller value of HD means better prediction performance. RVD denotes the volume difference between the predicted segmentation and the ground truth. The closer the value is to 0, the higher accuracy the model has. VOE stands for volume overlap error. The closer to 0, the better.
